# RAB27B Drives a Cancer Stem Cell Phenotype in NSCLC Cells Through Enhanced Extracellular Vesicle Secretion

**DOI:** 10.1158/2767-9764.CRC-22-0425

**Published:** 2023-04-17

**Authors:** Kayleah M. Meneses, Prita Pandya, Jennifer A. Lindemann, Dania S. Al-Qasrawi, Ryan A. Argo, Celeste M. Weems, Danielle J. Beetler, Geraldine V. Vijay, Irene K. Yan, Joy Wolfram, Tushar Patel, Verline Justilien

**Affiliations:** 1 Department of Cancer Biology, Mayo Clinic, Jacksonville, Florida.; 2 Mayo Clinic Graduate School of Biomedical Sciences, Jacksonville, Florida.; 3 University of North Florida, Jacksonville, Florida.; 4 Vivian l. Smith Department of Neurosurgery, McGovern School of Medicine, The UT Brown Foundation Institute of Molecular Medicine, The University of Texas Health Science Center, Houston, Texas.; 5 Department of Transplantation, Mayo Clinic, Jacksonville, Florida.; 6 School of Chemical Engineering/Australian Institute for Bioengineering and Nanotechnology, University of Queensland, Brisbane, Queensland, Australia.; 7 Comprehensive Cancer Center, Mayo Clinic, Jacksonville, Florida.

## Abstract

Cancer stem cells (CSC) within non–small cell lung carcinoma (NSCLC) tumors drive NSCLC progression, metastasis, relapse, and intrinsic chemoresistance. Understanding the mechanisms that support the malignant phenotypes of NSCLC CSCs may provide insights for improved NSCLC therapeutic interventions. Here, we report that expression of RAB27B, a small GTPase, is significantly upregulated in NSCLC CSCs when compared with bulk cancer cells (BCC). Short hairpin RNA–mediated knockdown of RAB27B leads to a loss of stem cell marker gene expression and reduced NSCLC spheroid growth, clonal expansion, transformed growth, invasion, and tumorigenicity. We find that NSCLC CSCs secrete significantly more extracellular vesicles (EV) than BCCs, and that this is RAB27B-dependent. Furthermore, CSC-derived EVs, but not BCC-derived EVs, induce spheroid growth, clonal expansion, and invasion in BCCs. Finally, RAB27B is required for CSC-derived EV-induced stemness in BCCs. Taken together, our results indicate that RAB27B is required for maintenance of a highly tumorigenic, cancer-initiating, invasive stem-like cell population in NSCLC and RAB27B is involved in propagating EV-mediated communication from NSCLC CSCs to BCCs. Our findings further suggest that inhibition of RAB27B-dependent EV secretion may be a potential therapeutic strategy for NSCLC.

**Significance:**

Expression of RAB27B in CSCs leads to elevated levels of EVs that mediate communication between CSCs and BCCs that maintains a stem-like phenotype in NSCLC cells.

## Introduction

Lung cancer is the second most diagnosed cancer and the leading cause of cancer deaths, accounting for 11.4% of total new cancer cases and 18% of total cancer deaths worldwide (1). The non–small cell lung carcinoma (NSCLC) subtype accounts for 80%–85% of lung cancer diagnoses and is further subclassified into lung adenocarcinoma (LUAD), squamous cell carcinoma (LUSC), and large-cell carcinoma. Despite recent developments in diagnostic methods and advances in immuno- and molecular-targeted therapies, the overall 5-year survival rate for patients with NSCLC remains at 10%–20% in most countries (1). Nearly all NSCLC deaths result from metastasis, recurrence, and/or chemoresistance (2). The high rate of NSCLC lethality and treatment failures has been attributed to subpopulations of cells within human NSCLC tumors that exhibit stem-like properties. These specialized cells, called cancer stem cells (CSC), exhibit elevated expression of well-established stem cell markers, the ability to self-renew, and a greater differentiation capacity to produce multilineage progenies (3–6). CSCs have been shown in various experimental models to possess enhanced promalignant properties, including tumor establishment and maintenance, aggressive invasion and metastasis, and enhanced resistance to radiation, chemotherapies, and immunotherapies, which contribute to relapse and poor outcome (3, 7–10). Thus, therapeutic strategies aimed at eliminating CSCs may improve the clinical prognosis of patients with NSCLC.

Extracellular vesicles (EVs) are membrane-bound nanoparticles released by cells that carry functional biomolecule cargos (proteins, carbohydrates, lipids, DNA, and RNA), between neighboring and distant cells. EVs represent an important mechanism of communication between CSCs and the surrounding tumor microenvironment (11). CSC-derived EVs can direct paracrine and autocrine signaling mechanisms that promote multiple aspects of tumor biology, including proliferation, invasion, metastasis, drug resistance, endothelial permeability, and remodeling of the tumor immune microenvironment (12–17). Given the importance of CSC-derived EVs in the biology of tumors, defining the mechanisms by which CSC-derived EVs promote a transformed phenotype can provide novel avenues for treatment of NSCLC.

RAB proteins are small GTPases that act as molecular switches that cycle between a GTP-bound active form and GDP-bound inactive form. In the active form, RABs recruit effector proteins and coordinate vesicle trafficking in cells, a process that is integral to many cellular processes (18). Rab GTPases localize in distinct subcellular organelles and govern specific vesicle transport pathways between different cellular compartments (18). Among the RAB GTPases, the RAB27 isoforms, RAB27A and RAB27B, play a key role in EV release by promoting the targeting of multivesicular endosomes (MVE) to the cell periphery and their docking at the plasma membrane (19). Interestingly, RAB27A/B has been shown to participate in nonredundant roles and signal to distinct effector proteins in the MVE pathway (20). Altered expression of RAB27A/B is observed in various human cancers and has been linked to cancer progression in NSCLC (21), although the mechanism by which RAB27B affects NSCLC is not known.

In this study, we used *in vitro* and *in vivo* methods to explore a mechanism by which CSCs induce EV production and assessed whether an enhanced release of EVs associates with an increased malignant phenotype in NSCLC CSCs. Our results demonstrate that RAB27B is upregulated in NSCLC CSCs and mediates an increase in EV release that propagates EV-mediated communication between CSCs and bulk cancer cells (BCC) that confers a stem-like phenotype in NSCLC cells.

## Materials and Methods

### Antibody Reagents and Cell Lines

The following antibodies were used: RAB27A (Cell Signaling Technology, catalog no. 69295, RRID:AB_2799759), α-Tubulin (Cell Signaling Technology, catalog no. 2144, RRID:AB_221054), CD9 (Cell Signaling Technology, catalog no. 13174, RRID:AB_2798139), ALIX (Cell Signaling Technology, catalog no. 2171, RRID:AB_2299455), and Calnexin (Cell Signaling Technology, catalog no. 2433, RRID:AB_2243887) cleaved caspase 3 (Cell Signaling Technology, catalog no. 9661, RRID:AB_2341188; Cell Signaling Technology); RAB27B (Proteintech, catalog no. 13412-1-AP, RRID:AB_2176732; Proteintech); CD81 (Santa Cruz Biotechnology, catalog no. sc-166029, RRID:AB_227589; Santa Cruz Biotechnology); TSG101 (catalog no. EXOAB-TSG101-1) from (Systems Biosciences); Ki67 (Abcam, catalog no. ab15580, RRID:AB_443209; Abcam); and CD31 (R&D Systems, catalog no. AF3628, RRID:AB_2161028; R&D Systems). Human NSCLC cell lines A549 (catalog no. CCL-185; RRID:CVCL_0023), H226 (catalog no. CRL-5826; RRID:CVCL_1544), H1299 (catalog no. CRL-5803; RRID:CVCL_0060), H1650 (catalog no. CRL-5883; RRID:CVCL_1483) were purchased from and grown as described by ATCC; PC9 cells (Sigma, catalog no. 90071810-1VL, RRID:CVCL_B260) were purchased from Sigma Millipore; and HARA cells (catalog no. JCRB1080.0, RRID:CVCL_2914) from Sekisui Xeno Tech, LLC. Cells were authenticated using short tandem repeat and monitored for *Mycoplasma* contamination using the Lonza MycoAlert *Mycoplasma* Detection Kit (Lonza). Cell lines were used no more than 10 weeks after thawing and passaged no more than 25 times. Cell growth media, F-12K Mixture Kaighn's modification, RPMI Medium 1640 and DMEM was purchased from Gibco Life Technologies (Life Technologies). Supplementary components FBS, Sodium pyruvate, Sodium bicarbonate, HEPES, l-Glutamine, and Pen Strep were purchased from Gibco Life Technologies. Prior to use, cell culture media was supplemented following the ATCC instructions specific for each cell type provided online (www.atcc.org).

### Enrichment and Clonal Expansion of NSCLC CSCs

CSCs were enriched from NSCLC cells by culturing 10,000 cells/mL in CSC medium consisting of serum-free DMEM-F12 medium (Gibco-Invitrogen) containing 50 μg/mL insulin (Sigma-Aldrich), 0.4% Albumin Bovine Fraction V (Sigma-Aldrich), N-2 Plus Media Supplement (R&D Systems), B-27 Supplement (Gibco-Invitrogen), 20 μg/mL EGF (PeproTech), and 10 μg/mL bFGF (PeproTech) in ultra-low attachment flasks (Corning). CSC cultures were expanded by trypsinization and mechanical dissociation followed by replating of single-cell suspensions (10,000 cells/mL) in fresh CSC medium. CSCs were collected for experiments after 2 weeks in nonadherent culture. For clonal expansion, single cells were added to each well of 96-well ultra-low attachment tissue culture plates (Corning) and clonal expansion was monitored at the indicated timepoints. For some experiments, the cells were incubated with 10^9^ or 10^10^ EVs/mL (EVs from conditioned media of A549 or H1299 BCC and CSCs isolated as described below) and fresh EVs were added every 5 days during the course of the experiment. CSC sphere diameters were determined using Image-Pro Plus 7 (Image-Pro Plus, RRID:SCR_016879; Media Cybernetics).

### RNA Isolation, qPCR, and Immunoblot Analysis

Total RNA was extracted from NSCLC cells using the RNeasy Plus Mini Kit (Qiagen). qPCR reagents for *RAB27B* and stemness gene mRNAs were purchased from Applied Biosystems and are listed in Supplementary Table S1. qPCR was carried out using an Applied Biosystems ViiA7 thermal cycler, and data were analyzed using the SDS 2.3 software package. Data were normalized to Ubiquitin C mRNA. The bicinchoninic acid assay kit was used to determine protein concentration for sample lysates according to the manufacturer's instructions (Thermo Fisher Scientific).

### Lentiviral RNAi Constructs and Cell Transduction

Lentiviral RNAi against human *RAB27B* were obtained from Sigma-Aldrich Mission short hairpin RNA (shRNA) library and packaged into recombinant lentiviruses as described previously (22). A nontarget lentiviral shRNA (shNT) that does not recognize any human or mouse genes was used as a negative control. shRNA target sequences are listed in Supplementary Table S1. Transduced stable cell populations were generated as described previously (22). shRNA constructs were analyzed for efficiency of RAB27B knockdown by qPCR and immunoblot analysis as described previously (23).

### Soft Agar Growth and Cellular Invasion Analysis

Anchorage-independent growth was assessed by the ability of cells suspended in agarose to form colonies (SeaPlaque GTG Agarose from Lonza). Complete 2X media was mixed with 1.5% agarose at 1:1 ratio to achieve a final agar concentration of 0.75% in growth media plated into 35 mm tissue culture dishes to create a bottom layer of soft agar. Single-cell suspensions (either BCCs or CSCs) containing 5,000 cells per plate were mixed in soft agar and dispensed over the solidified bottom layer of soft agar. Plates were incubated at 37°C in 5% CO_2_ and colony growth was assessed after 4–5 weeks following staining with Giemsa (EMD Millipore). Briefly, plates were fixed in methanol for 20 minutes followed by two 1X PBS washes. Giemsa stain was diluted (1:20) in 1X PBS and fixed colonies were stained at room temperature for 2 hours. Stained plates were washed with 1X PBS then imaged using BioSpectrum (UVP). Colony size and number were determined using Image-Pro Plus version 7 (Image-Pro Plus, RRID:SCR_016879) software.

Cellular invasion was measured in 24-well plate transwell chambers containing inserts coated with Matrigel basement membrane (Corning Costar) as described previously (22). A total of 1 × 10^5^ cells (either BCCs or CSCs) were seeded in serum-free media into the upper chamber of the transwell assay. A total of 750 μL culture media supplemented with 2.5% FBS was added in the lower chamber. For some experiments, the cells were incubated with 10^10^ EVs/mL for 24 hours before and during invasion assays. After 18 hours, transwell chambers were fixed with methanol and stained with crystal violet to detect invaded cells. Images of invaded cells were captured using an Olympus microscope and quantitated using Image-Pro (Image-Pro Plus, RRID:SCR_016879) software.

### Three-dimensional Cultures

NSCLC CSCs were clonally expanded for 1 week. Spheres were gently suspended in 20% Matrigel and were seeded into 96-well plates onto a layer of 35 μL of Matrigel Growth Factor Reduced Basement Membrane Matrix (BD Biosciences). Once solidified, CSC culture medium was placed on top of the cells. Spheres were visualized and images were captured microscopically (Olympus microscope) 5 days after plating and photographed to observe cellular morphology.

### Tumorigenicity in NOD-SCID Mice and IHC Analysis

All animal experiments were performed under the approved Mayo Clinic Institutional Animal Care and Use Committee protocol (A00005139-20). A total of 1 × 10^6^ PC9 *shNT* and *shRAB27B* cells were suspended in 50% Growth Factor Reduced Matrigel Matrix (BD Biosciences) in 1X PBS and subcutaneously injected into the right flank of NOD-SCID mice purchased from Jackson laboratory using a 28-gauge needle in a final volume of 50 μL as described previously (23). Tumor growth was monitored by caliper measurement and tumor volume was estimated using the formula 0.5236 (*L* × *W* × *H*), where *L* represents the length of the tumor, *W* represents the width of the tumor, and *H* represents the height as described previously (24). After 8 weeks, mice were harvested, tumors collected, and final tumor weights were determined.

In a separate cohort of NOD-SCID, mice were injected with 2.5 × 10^6^ A549-luciferase *shNT* or *shRAB27B* cells in 100 μL of 1X PBS through the tail vein. For *in vivo* imaging, mice were anesthetized with isoflurane and injected intraperitoneally with d-luciferin (15 mg/kg) and observed using an IVIS system (Caliper Life Sciences). After 8 weeks, mice were euthanized and lungs harvested to evaluate for metastatic tumor formation.

For IHC analysis, harvested subcutaneous xenograft flank tumors or lungs were formalin-fixed, embedded in paraffin, serially sectioned, and stained with hematoxylin and eosin (H&E) as described previously (25). H&E-stained sections of lungs were imaged using Aperio ScanScope XT and analyzed using Aperio ImageScope (v11.1.2.752) to determine tumor number and burden. Subcutaneous flank tumor sections were IHC stained for Ki67 (Dako), cleaved caspase 3 (Cell Signaling Technology), and CD31/PECAM1 (Santa Cruz Biotechnology) using antibodies diluted in PBS/Tween and visualized using Envision Plus Dual Labeled Polymer Kit following the manufacturer's instructions (Dako). Images were captured and analyzed using Aperio ScanScope scanner and software (Aperio Technologies).

### EV Isolation

For EV isolation, A549 and H1299 adherent BCCs were seeded in 150 mm dishes with growth media supplemented with 10% EV-depleted FBS (System Biosciences) and CSCs were seeded in T75 low adherence flask with EV-free CSC media (serum-free DMEM-F12 medium; Gibco-Invitrogen) containing 0.4% Albumin Bovine Fraction V (Sigma-Aldrich), B-27 Supplement (Gibco-Invitrogen), 20 μg/mL EGF (PeproTech), and 10 μg/mL bFGF (PeproTech). The media was collected after 48 hours and centrifuged at 300 × *g* for 10 minutes at 4°C. The supernatant was transferred to a new polypropylene tube and centrifuged at 2,000 × *g* for 20 minutes at 4°C. The supernatant then was filtered through a 0.22 μm polyethersulfone membrane filter (Corning Incorporated) and transferred to a polyallomer ultracentrifuge tube and centrifuged at 10,000 × *g* for 30 minutes at 4°C (Beckman Coultier Optima Max Ultra). The supernatant was then transferred to a new polyallomer ultracentrifuge tube and centrifuged at 100,000 × *g* for 70 minutes at 4°C (Beckman Coultier Optima Max Ultra). The pellet was then suspended in 1X PBS, and the ultracentrifugation step was repeated to obtain an EV pellet that was suspended in 200 μL of sucrose buffer (5% sucrose, 50 mmol/L Tris, and 2 mmol/L MgCl).

### Nanoparticle Tracking Analysis

The size and concentration of isolated EVs were determined by nanoparticle tracking analysis (NTA). EVs were diluted (1:100) in 1X PBS and analyzed (1 mL) with a NanoSight LM10 (Malvern Panalytical; 60 seconds measurement; three capture replicates).

### Transmission Electron Microscopy

EVs were fixed in 4% paraformaldehyde (1:1) at room temperature (2 minutes), placed (2 μL) on carbon-formvar-coated copper grids (300 mesh), and blotted. Water (2 μL) was added to the samples that were then blotted, and 2% aqueous uranyl acetate (2 μL) was placed on the grid (2 minutes) and blotted. The grids were examined with a 208S Electron Microscope (FEI; 60 kV, Philips). Digital images were obtained with an 831 Orius Camera (Gatan).

### Fluorescent Labeling of EVs, Uptake, and Flow Cytometry

EVs were labeled using DiI (Invitrogen) following the manufacturer's protocol. A total of 5 × 10^3^ BCCs were cultured overnight on each well of glass 8-well cell chamber slides. BCCs were incubated with DiI-labeled EVs for 6 hours. Cells were stained with Phalloidin-Alexafluor 488 and 4′,6-diamidino-2-phenylindole (Thermo Fisher Scientific) for visualization of F-actin filaments and nuclei, respectively. The cells were mounted with Aqua-Poly/Mount (Polysciences, Inc.). Images were acquired using a fluorescent microscope (IX71 Olympus). For flow cytometry analysis, BCCs were incubated with 10^4^ BCC or CSC-derived EVs/cell for 6 hours in EV-free media. BCCs were collected, pelleted, resuspended in PBS, stained with Sytox blue (Thermo Fisher Scientific) for 15 minutes at room temperature, and analyzed by flow cytometry (Attune NxT flow cytometer; Thermo Fisher Scientific).

### Statistical Analysis

All *in vitro* results were confirmed in at least three independent experiments. Statistical significance of results was analyzed using the GraphPad Prism (GraphPad Prism, RRID:SCR_002798) program version 9. The statistical differences were analyzed using the Student paired two-tailed *t* test, one-way ANOVA, and *χ*^2^ analysis. *P* values less than 0.05 were considered significant.

### Data Availability

The data generated in this study are available within the article and its Supplementary Data or upon request from the corresponding author.

## Results

### NSCLC CSCs Express Elevated RAB27B

**FIGURE 1:**
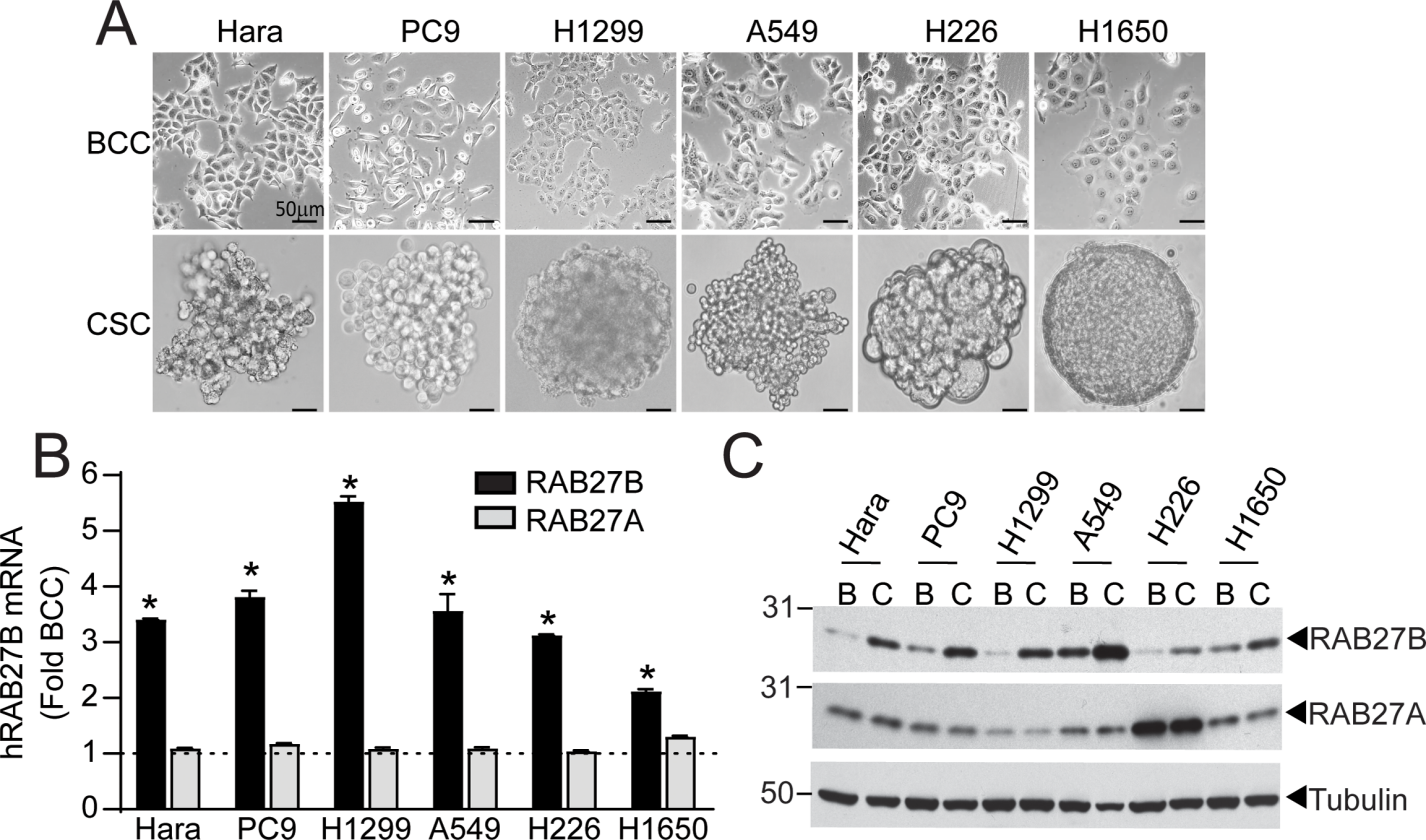
RAB27B expression is elevated in NSCLC CSCs. **A,** Photomicrographs of a panel of NSCLC BCCs grown in adherent culture (top) and CSCs cultured in low-adherence culture (bottom). Scale bar, 50 μm. **B,** qPCR for *RAB27B* and *RAB27A* mRNA abundance in BCC and CSCs. Results are presented as fold of BCC ± SEM; *n* = 3; *, *P* < 0.05 compared with BCC. Dashed line at one represents BCC-normalized value. **C,** Immunoblot analysis showing expression of RAB27A and RAB27B in BCCs (B) and CSCs (C). Tubulin was used as a loading control.

CSCs can be functionally enriched by culturing the heterogeneous population of BCCs in serum-free defined media under ultra-low adherence culture conditions (4, 6). These conditions favor growth of highly tumorigenic stem-like cells, while negatively selecting for less tumorigenic differentiated tumor cells. We have demonstrated that NSCLC CSCs isolated using this method exhibit hallmark properties of CSCs, including spheroid growth with long-term repopulation potential, clonal expansion, enhanced transformed growth and tumor initiation, and a stem-like transcriptional profile (6). To uncover novel drivers of stemness in NSCLC cells, we previously performed RNA sequencing (RNA-seq) to identify genes differentially expressed between NSCLC BCCs and CSCs (6). Our analysis identified *RAB27B* as significantly upregulated in the CSCs compared with BCCs. To validate these results, we isolated CSCs from a panel of established NSCLC cell lines (Hara, PC9, H1299, A549, H226, H1650) that represent the two major forms of NSCLC, LUAD, and LUSC, and compared their RAB27B expression with BCCs grown under adherent culture conditions (Fig. 1A). qPCR analysis showed that *RAB27B* mRNA abundance was significantly induced in CSCs compared with BCCs with relative increases in the range of 2- to 5-fold (Fig. 1B). Immunoblotting confirmed that RAB27B protein was overexpressed in the CSCs (Fig. 1C). Interestingly, qPCR and immunoblot analysis revealed that the expression of the closely related RAB27A isoform was not induced (Fig. 1B and C) suggesting that induction of RAB27B was isoform specific in NSCLC CSCs. To determine whether these observations recapitulate in primary human NSCLC cells, we next analyzed *RAB27B* mRNA abundance in stem-like cultures established from primary human NSCLCs obtained from patients undergoing tumor resection (6). Similar to established NSCLC cell lines, primary NSCLC CSC cultures expressed elevated *RAB27B* mRNA levels when compared with the bulk tumor cells from which they were isolated (Supplementary Fig. S1A). To determine whether RAB27B expression is more generally induced in NSCLC CSCs, we extended our analysis to the stem-like population of mouse NSCLC tumors. Consistent with human NSCLC, CSCs isolated from cell lines established from LUAD tumors that developed in the *Kras^G12D^;Trp53^fl/fl^* (KP) mouse model (26) showed elevated RAB27B mRNA expression (Supplementary Fig. S1B). Thus, CSCs isolated from NSCLC cells of multiple origins express elevated levels of *RAB27B* compared with bulk NSCLC cells, suggesting that RAB27B is targeted for overexpression in CSCs.

### NSCLC CSCs Require RAB27B for Clonal Expansion and Anchorage-independent Growth

**FIGURE 2:**
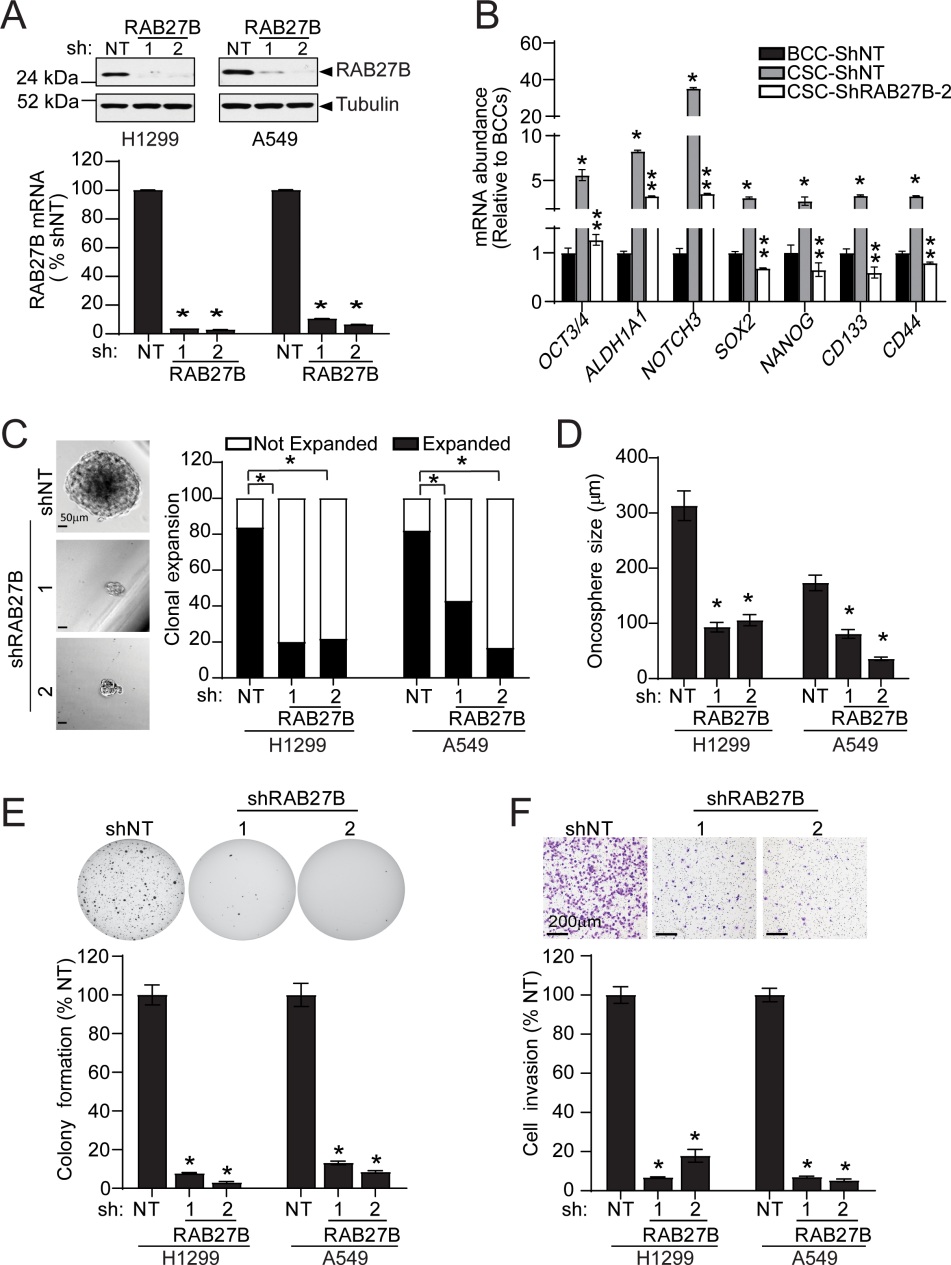
RAB27B is required for the stem-like phenotype of NSCLC CSCs. **A,** qPCR and immunoblot analysis of RAB27B mRNA and protein abundance in H1299 and A549 *shRAB27B* knockdown CSCs. **B,** qPCR for stem cell markers in *shNT* BCC, and *shNT* and *shRAB27-2* A549 CSCs. Represented as mean ± SEM; *n* = 3; *, *P* < 0.05 *shNT* BCC versus *shNT* CSC and **, *P* < 0.05 *shNT* CSC versus *shRAB27-2*. **C,** Photomicrographs of single H1299 and A549 *shNT* and *shRAB27B* CSC spheres and clonal expansion efficiency of H1299 and A549 CSCs in nonadherent culture. Results presented as % of single CSCs that expanded or did not expand; *, *P* < 0.05 compared with NT. *n* = 40 (H1299) and 41 (A549). **D,** Sphere size expressed as mean diameter in micrometers ± SEM; *n* = 40 (H1299), and 41 (A549); *, *P* < 0.05 compared with *shNT*. **E,** Representative photomicrographs showing soft agar colony formation and quantitation of soft agar colonies formed by A549 and H1299 *shNT* and *shRAB27B* CSCs. Results represent the mean ± SEM; *n* = 5; *, *P* < 0.05 compared with NT. **F,** Representative photomicrographs of A549 and H1299 *shNT* and *shRAB27B* CSC cellular invasion through Matrigel-coated chambers. Quantitated results are expressed as % NT control and represent the mean ± SEM; *n* = 4; *, *P* < 0.05 compared with NT control.

Our observation of elevated *RAB27B* expression in stem-like cells isolated from both NSCLC established cell lines and primary tumors suggested that RAB27B may be important for the maintenance of NSCLC CSCs. To directly assess the requirement of RAB27B, we performed shRNA-mediated knockdown of *RAB27B* in a panel of NSCLC CSCs (H1299. A549, Hara and PC9) utilizing two independent *RAB27B* shRNAs (*shRAB27B*; Supplementary Table S1). CSCs expressing a nontargeting shRNA (*shNT*) were used as control cells. The efficiency of *RAB27B* mRNA and protein knockdown were confirmed by qPCR and immunoblot analysis, respectively (Fig. 2A; Supplementary Fig. S2A). qPCR analysis for expression of well-characterized stemness genes revealed that A549 CSC cultures express elevated mRNA for many genes associated with the stem cell phenotype (27) including OCT3/4, ALDH1A1, NOTCH3, SOX2, NANOG, CDC133, and CD44 when compared with A549 BCC cultures (Fig. 2B; Supplementary Fig. S2B). Interestingly, we observed a decrease in the expression of these stemness genes upon knockdown of RAB27B in CSCs (Fig. 2B; Supplementary Fig. S2B). Given the loss of stemness markers upon RAB27B knockdown, we next examined whether RAB27B is important for NSCLC CSC self-renewal using clonal expansion assays. *shNT* or *shRAB27B* CSCs were plated as single cells in individual wells of ultra-low adherence 96-well culture plates and their ability to clonally expand into spheroids was followed over a 2-week period. Quantitative analysis of individual clones demonstrated that *shNT* CSCs exhibited a very high clonal expansion efficiency (∼84% for H1299, ∼84% for A549, ∼63% for Hara, ∼86% for PC9), whereas shRAB27B CSCs from all four cell lines exhibited a significant reduction in clonal expansion efficiency (∼5%–11% for H1299, ∼33%–43% for A549, ∼23%–31% for Hara, ∼24%–30% for PC9; Fig. 2C; Supplementary Fig. S2C). *shNT* CSCs expanded into large spheroids, whereas the majority of *shRAB27B* CSCs formed smaller spheroids (Fig. 2C) which was confirmed by quantitation of expanded spheroid size (Fig. 2D; Supplementary Fig. S2D). Thus, RAB27B plays a critical role in NSCLC CSC self-renewal, a hallmark characteristic of CSCs.

Anchorage-independent growth is a characteristic attributed to the stem-like cell population within cancer cells. Soft agar colony formation is a well-established method to measure anchorage-independent growth and it has been demonstrated that cancer cell colonies formed in agar-based three-dimensional (3-D) culture exhibit traits of stemness (28). To evaluate the role of RAB27B in anchorage-independent growth exhibited by CSC cultures, we performed soft agar assays on *shNT* and *shRAB27B* CSCs. Knockdown of *RAB27B* significantly inhibited soft agar colony formation in CSCs when compared with *shNT* CSCs (Fig. 2E; Supplementary Fig. S2E). Consistent with previous observations (6, 29), we found that NSCLCs CSCs had an enhanced ability to grow as soft agar colonies when compared with BCC cultures (Supplementary Fig. S2F). Notably, soft agar colony formation was also impaired in *shRAB27B* BCCs (Supplementary Fig. S2F), suggesting that RAB27B is important for the maintenance of a CSC population within BCCs.

### NSCLC CSCs Require RAB27B for Invasion

Cancer metastasis involves tumor cell invasion through a dense extracellular matrix. CSCs have been shown to have a higher propensity to invade in a variety of cancers (30, 31). Therefore, we next investigated whether *shNT* and *shRAB27B* CSCs differ in invasive ability. For this purpose, isolated H1299 *shNT* and *shRAB27B* CSC spheroids were plated in 3-D Matrigel cultures and the morphology of spheroids was examined 5 days later. *shNT* CSCs generated prominent cellular protrusions extending from the spheroids and invading into the surrounding matrix (Supplementary Fig. S2G), morphology consistent with a highly invasive phenotype (32). In contrast, *shRAB27B* CSC spheroids exhibited rounded morphology with few cellular projections, suggesting a less invasive phenotype (Supplementary Fig. S2G). To further assess the requirement of RAB27B in CSC invasive behavior, we performed Boyden chamber assays. Quantitation of invaded cells revealed that *shNT* CSCs readily invaded through the Matrigel-coated transwell chambers, whereas *shRAB27B* CSCs exhibited a significantly reduced invasive potential (Fig. 2F; Supplementary Fig. S2H). Together, these data show that RAB27B is required for multiple hallmarks of CSC behavior including the expression of stemness-associated genes, clonal expansion, transformed growth, and cell invasion.

### NSCLC CSCs Exhibit RAB27B-dependent Tumor-initiating Activity *In Vivo*

**FIGURE 3:**
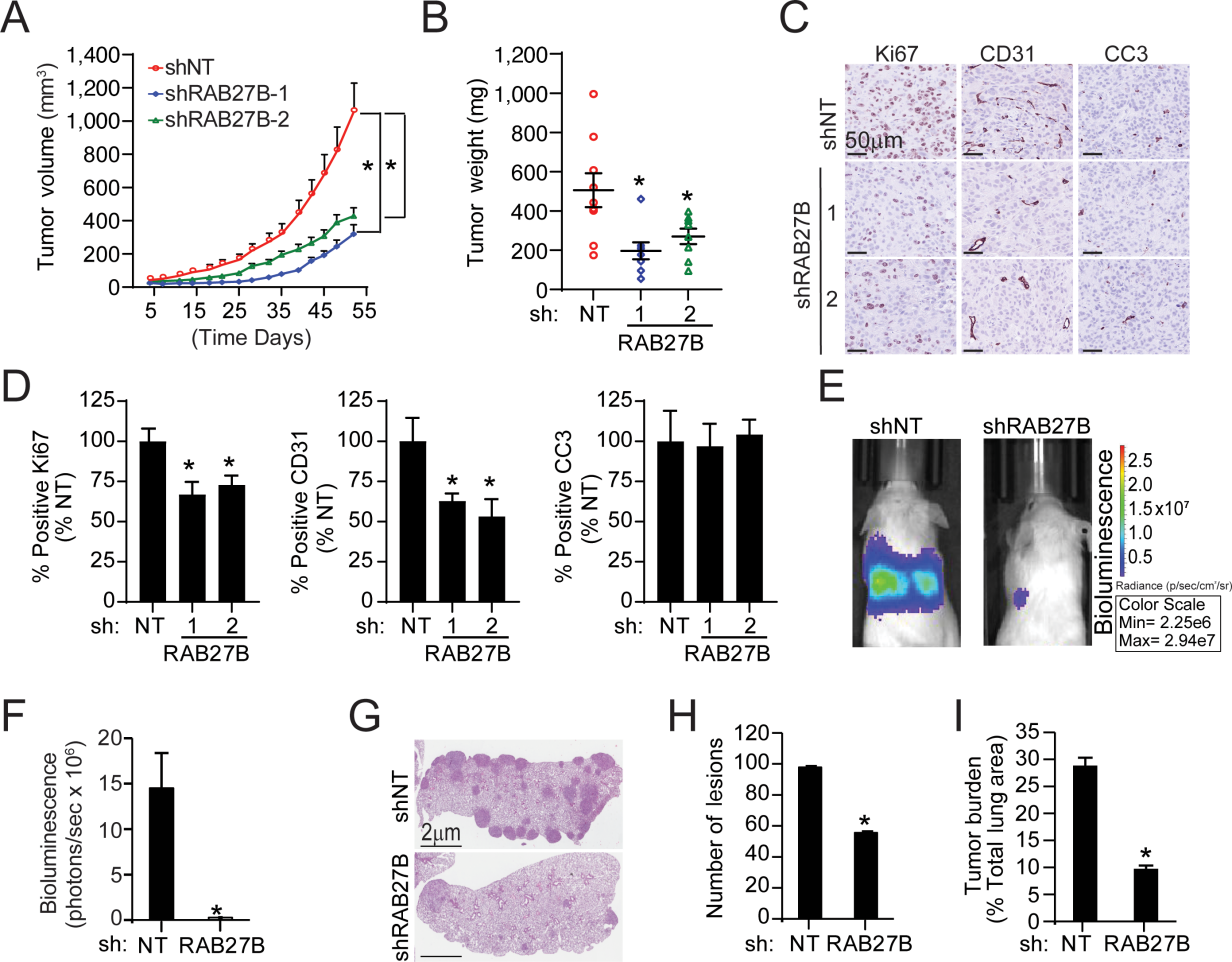
RAB27B is required for NSCLC tumorigenicity *in vivo*. **A,** Growth curves of PC9 *shNT* and *shRAB27B* CSCs as subcutaneous flank tumors. **B,** Final tumor weight of PC9 *shNT* and *shRAB27B* CSC tumors at endpoint. **C,** Representative photographs of Ki67, CD31, and cleaved caspase 3 (CC3) staining in PC9 *shNT* and *shRAB27KD* CSC tumors. **D,** Quantitative analysis of IHC staining in C. Data in A, B, and D presented as mean ± SEM; *n* > 5 mice/group; *, *P* < 0.05 compared with *shNT*. **E,** Representative dorsal view of bioluminescent images of A549 *shNT* and *shRAB27B* CSC tumor-bearing mice. **F,** Quantitation of bioluminescence of orthotopic tumors developed in the lungs of mice. **G,** Representative images of H&E-stained lung sections from mice in E. Tumor size (**H**) and tumor burden (**I**) were assessed in at 8 weeks after CSC inoculation. Results in F, H, and I represent mean ± SEM; *n* = 4/group; *, *P* < 0.05 compared with *shNT* cells.

CSCs are particularly characterized by their enhanced tumorigenicity *in vivo*. Given the requirement of RAB27B for transformed behavior *in vitro*, we assessed the role of RAB27B in NSCLC tumorigenicity *in vivo*. *shNT* and *shRAB27B* PC9 cells were injected subcutaneously into the flanks of NOD-SCID mice and tumor growth was monitored over 8 weeks. PC9 cells were chosen because they grow as subcutaneous tumors with well-established growth kinetics (33). *shRAB27B* PC9 tumors grew at a slower rate than *shNT* PC9 tumors, resulting in significantly smaller tumors (Fig. 3A and B; Supplementary Fig. S3A). To gain insight into the basis for the reduced tumor formation by shRAB27B cells, we next examined the tumors histologically. IHC analysis confirmed that reduced RAB27B expression persisted in *shRAB27B* tumors (Supplementary Fig. S3B). At endpoint, *shRAB27B* tumors exhibited an approximate 30% reduction in Ki67 staining, a measure of cell proliferation, when compared with *shNT* tumors (Fig. 3C and D). In contrast, no significant difference was observed in cleaved caspase 3 staining, a marker of apoptosis (Fig. 3C and D), suggesting that the reduced growth of *shRAB27B* CSCs as tumors is due to their decreased proliferation. Angiogenesis also plays an important role in tumor cell proliferation. Therefore, we also assessed *shNT* and *shRAB27B* tumors for CD31 expression, a marker of angiogenesis. CD31 immunostaining was significantly decreased by approximately 40% in *shRAB27B* tumors compared with *shNT* tumors, indicating that RAB27B in the CSCs is important for tumor angiogenesis (Fig. 3C and D).

In a second *in vivo* tumor model, we injected *shNT* or *shRAB27B* A549 cells into the tail vein of NOD-SCID mice to assess lung colonization and growth as tumors. Cells additionally expressed luciferase to monitor formation of lung tumors by bioluminescent imaging. Our analysis revealed that *shRAB27B* A549 cells exhibited a significantly lower bioluminescence signal in the lung, suggesting their ability to colonize the lungs of mice and grow as tumors is impaired (Fig. 3E and F). After 8 weeks, the lungs of the inoculated mice were harvested for histologic analysis to validate results of bioluminescence imaging. Lungs from mice injected with *shNT* A549 cells were confirmed to have numerous tumor nodules, whereas mice inoculated with *shRAB27B* A549 cells had fewer and smaller nodules resulting in a lower tumor burden (Fig. 3G–I). These results demonstrate that reduced RAB27B expression decreased tumor growth of NSCLC CSCs.

### NSCLC CSCs Release Elevated Levels of EVs

**FIGURE 4:**
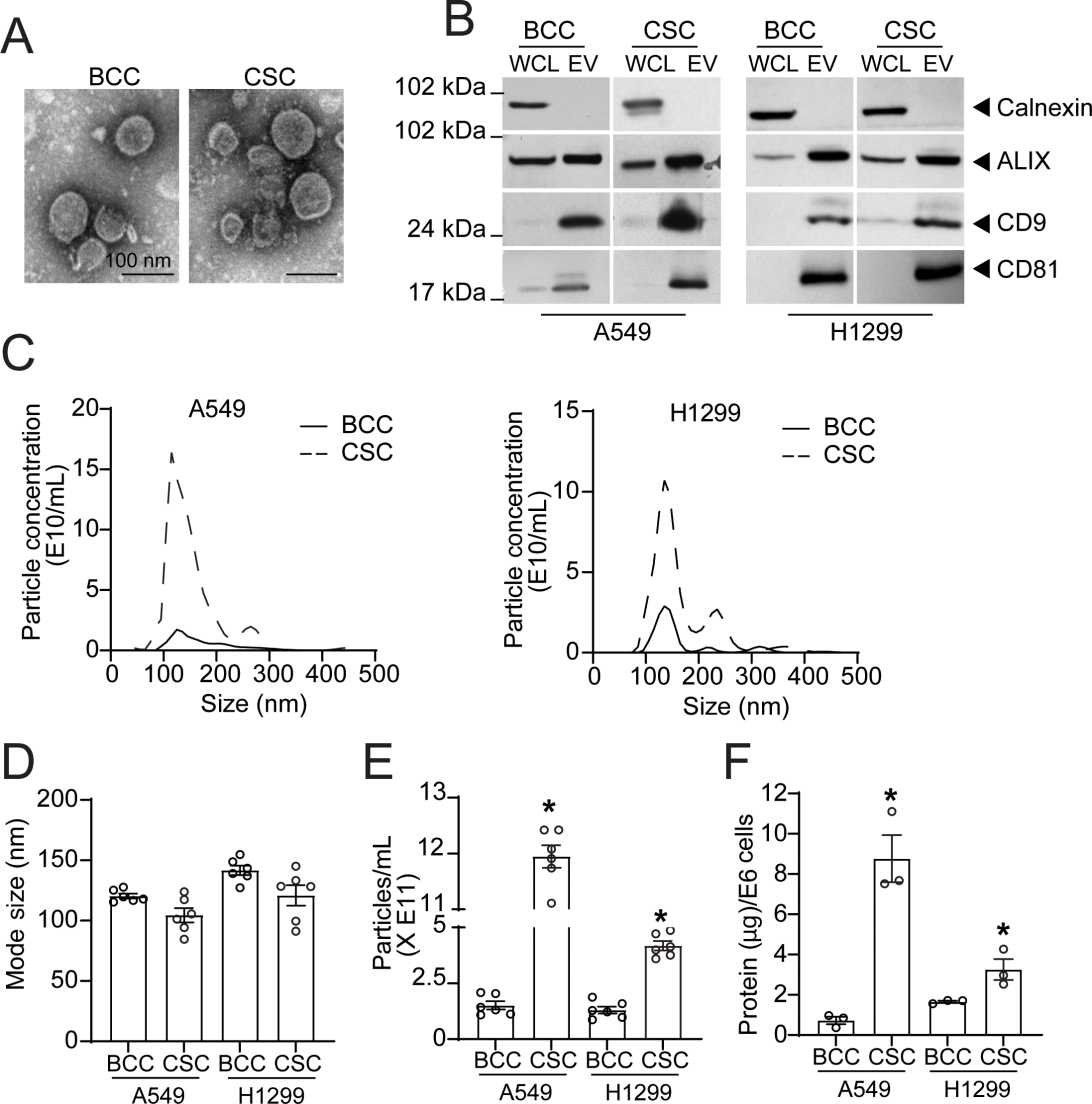
Characterization of EVs isolated from NSCLC BCCs and CSCs. **A,** TEM images showing the particle size and spherical vesicle morphology of BCC and CSC EVs (100 nm). **B,** Expression of calnexin, ALIX, CD9, and CD81 in the whole-cell lysate (WCL) and EVs of BCCs and CSCs by immunoblotting. NTA of EV mean particle concentration (**C**), mode size (**D**), mean number of particles isolated from 48-hour conditioned media (**E**), and total protein isolated from A549 and H1299 BCCs and CSCs (**F**). Data are presented as mean ± SEM from independent biological replicates *n* = 5 in D and E and (*n* = 3) in F. *, *P* < 0.05.

Our findings reveal that RAB27B plays an important role in the malignant behavior of NSCLC CSCs. In addition, RAB27B plays a required role in EV secretion (19). Therefore, we reasoned that RAB27B mediates the aggressive behavior of NSCLC cells through pathways that likely involve EVs. To investigate the release of EVs from NSCLC BCC and CSCs, we used differential ultracentrifugation to isolate EVs from conditioned media of A549 and H1299 BCCs and CSCs. Transmission electron microscopy (TEM) examination revealed that BCC- and CSC-derived EVs exhibited a similar membranous morphology that is characteristic of purified EV particles (Fig. 4A). Immunoblot analysis showed that both BCC- and CSC-derived EV preparations were enriched in the known EV markers, ALIX, CD9, and CD81 when compared with parental whole-cell lysates (Fig. 4B). The purity of isolated EVs was verified by immunoblotting for calnexin, an endoplasmic reticulum protein and a marker of intracellular vesicle contamination which was detected in whole-cell lysates, but not in EV lysates (Fig. 4B). Nanoparticle tracking analysis (NTA) was performed to determine size distribution and concentration of EV particles released from BCCs and CSCs (Fig. 4C). EV size distribution was consistent with published reports (34, 35). BCC-derived EVs showed a larger mode when compared with CSC-derived EVs (120.4 and 104.5 nm for A549 BCC- and CSC-derived EVs respectively, and 141.6 and 120.9 nm for H1299 BCC- and CSC-derived EVs respectively); however, this difference in mode was not statistically significant (Fig. 4D). We observed a marked increase in the number of EVs obtained from CSCs compared with BCCs (∼13- and ∼3-fold for A549 and H1299 cells, respectively; Fig. 4E). This difference in the number of EVs released was also reflected in EV protein concentration (protein per 10^6^ cells), which was higher in CSC EV isolates than in BCC EV isolates (Fig. 4F). Taken together, our data reveal that BCC- and CSC-purified EV particles exhibit similar physical and biochemical properties that are characteristic of EVs but show marked differences in the amount of EVs produced by equivalent numbers of cells.

### Elevated RAB27B Expression Associates with Increased EV Release in NSCLC CSCs

**FIGURE 5:**
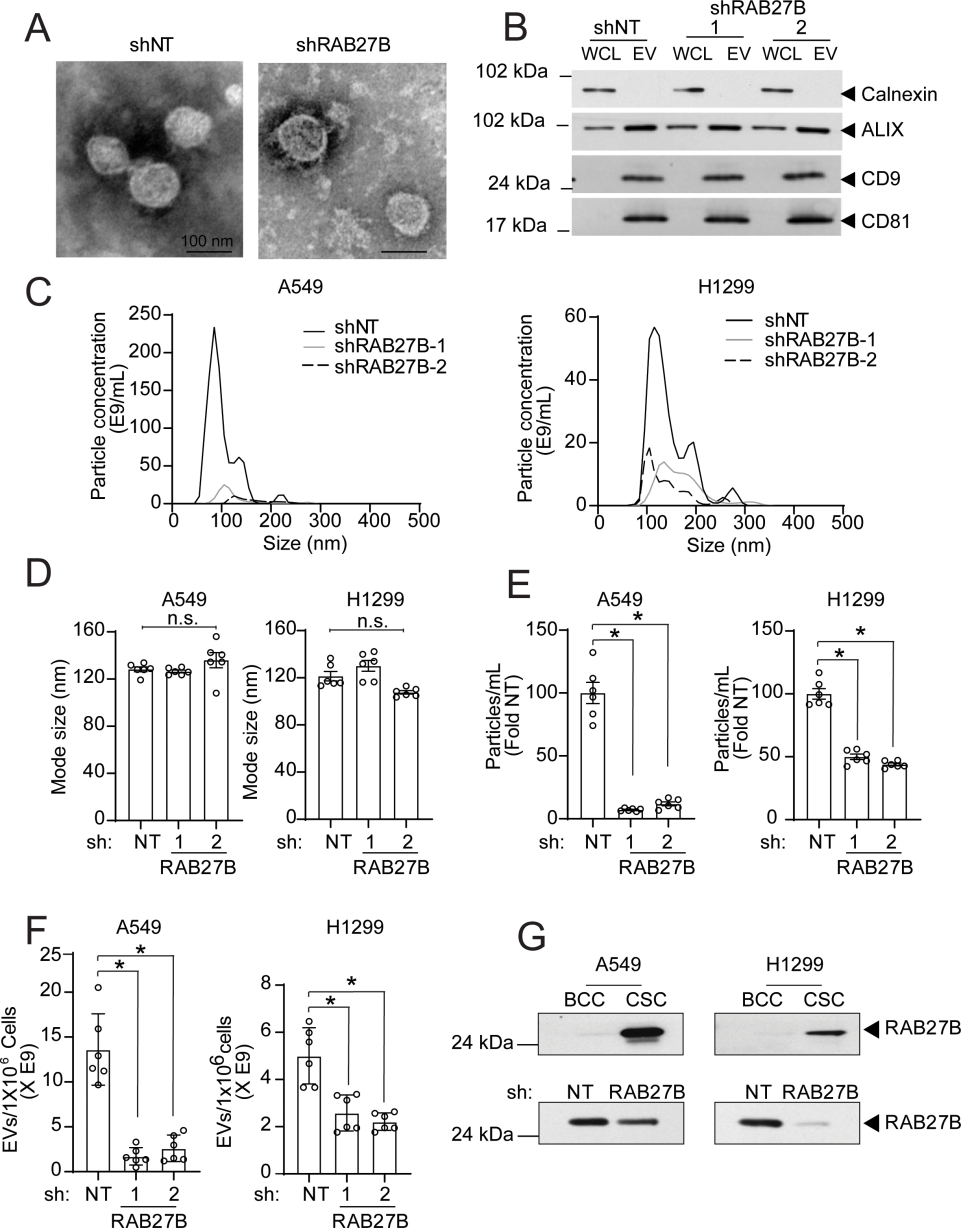
Characterization of RAB27B knockdown NSCLC CSC-derived EVs. **A,** TEM images showing the particle size and spherical vesicle morphology of A549 *shNT* and *shRAB27B-2* CSC-derived EVs (100 nm). **B,** Immunoblot analysis of calnexin, ALIX, CD9, and CD81 in the whole-cell lysate (WCL) and EVs of A549 *shNT* and *shRAB27B* CSC. NTA of *shNT* and *shRAB27B* A549 and H1299 CSC-derived EV size distribution (**C**), particle mode size (nm; **D**), particle concentration (**E**), and mean number of particles per 1 million cells (**F**). **G,** Immunoblot of RAB27B expression in BCC and CSC-derived EVs (top) and RAB27B expression in CSC *shRAB27B* versus *shNT*-derived EVs (bottom). Data in D–F are presented as mean ± SEM; *n* = 6; *, *P* < 0.05.

Our results show that CSCs express elevated RAB27B expression, exhibit *RAB27B*-dependent transformation, and release increased levels of EVs. Therefore, we next assessed whether the increased level of EVs in CSCs is dependent on RAB27B. We purified EVs secreted by *shNT* and *shRAB27B* CSCs and characterized EVs by TEM, immunoblot analysis, and NTA. TEM showed that EVs secreted by *shNT* and *shRAB27B* CSCs exhibited similar morphology (Fig. 5A). The EV markers, CD9, CD81, and ALIX were present at similar levels when equivalent amounts of EV protein from *shNT* and *shRAB27B* CSCs were analyzed by immunoblot analysis (Fig. 5B). Similar results were observed in H1299 CSCs (Supplementary Fig. S4A and S4B). Furthermore, stable knockdown of *RAB27B* had no impact on the size distribution (Fig. 5C) and mode (Fig. 5D) of EVs when compared with *shNT* cells. However, A549 and H1299 *shRAB27B* CSCs showed a 91% and 53% reduction in the amount of EVs released, respectively (Fig. 5E). Given that *shRAB27B* CSCs grow slower than *shNT* CSCs, we quantitated EV particles released on the basis of cell numbers. Our results demonstrated that *shRAB27B* cells released significantly fewer EVs per cell than *shNT* control cells (Fig. 5F). Because the RAB GTPases may be packaged as cargo in EVs (36), we next assessed for the presence of RAB27B in purified secreted EVs. Immunoblot analysis showed an increase in RAB27B detected in CSC-derived EVs when compared with BCC-derived EVs (Fig. 5G). Furthermore, the level of EV packaged RAB27B levels was decreased in *shRAB27B* knockdown CSCs (Fig. 5G). Taken together, our data show that NSCLC CSCs secrete elevated levels of EVs in a RAB27B-dependent manner.

### CSC EVs Increase the Aggressiveness of BCCs

**FIGURE 6:**
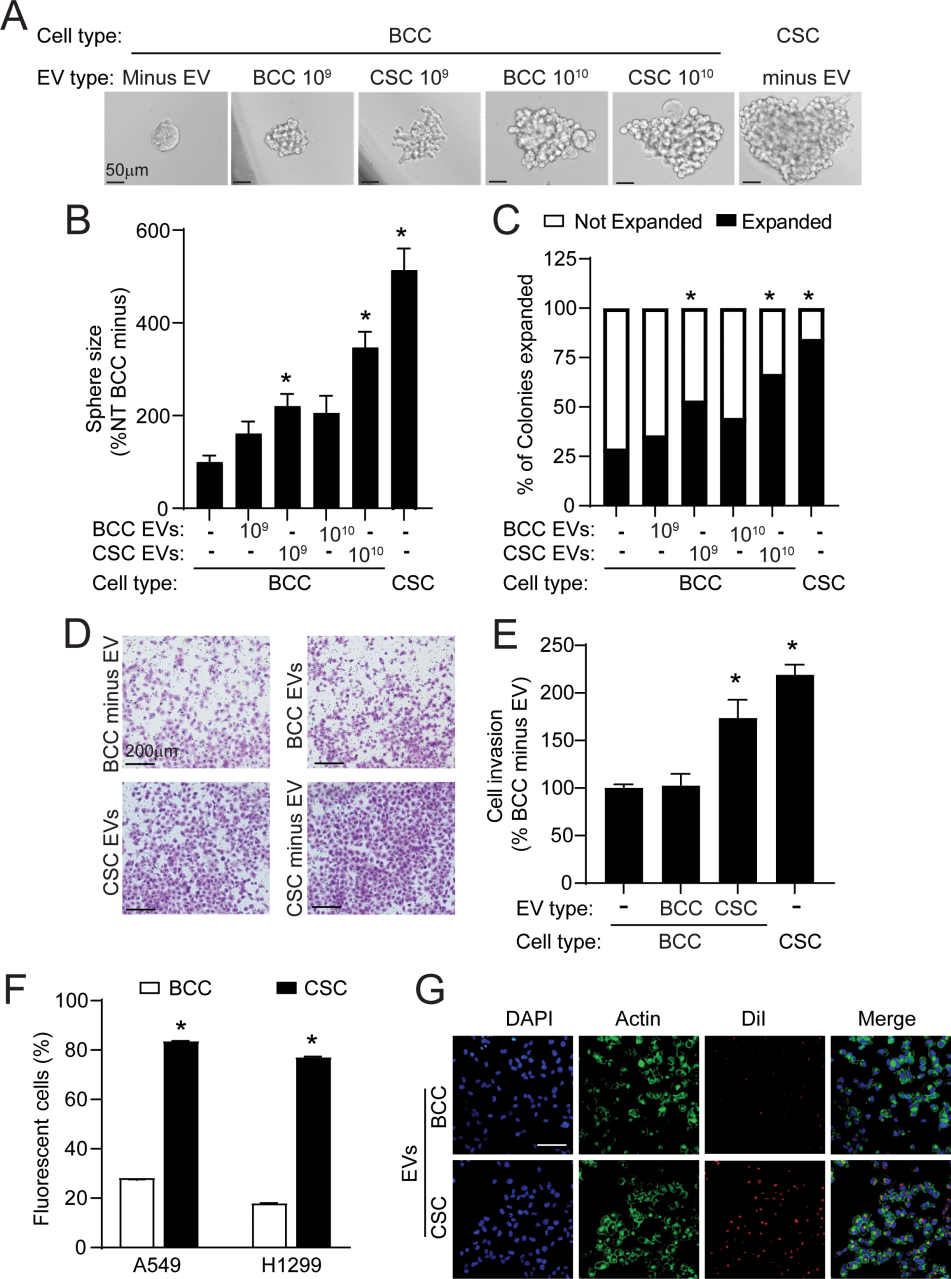
CSC EVs increase the aggressiveness of BCCs. **A,** Representative photomicrographs of expanded BCC spheres treated with 10^9^/mL or 10^10^/mL BCC or CSC EVs in low-adherent culture. Untreated BCC and CSCs served as controls. **B,** Quantitation of expanded sphere size expressed as percent of BCC minus EV. **C,** Quantitation of the percent of single cells that expanded or did not expand under each condition. For B and C, *n* = 51; *, *P* < 0.05 compared with BCC minus EV. **D,** Representative photomicrographs of A549 BCC invasion through Matrigel-coated chambers with 10^10^/mL BCC or CSC EVs. Untreated BCC and CSCs served as controls. **E,** Quantitated results of D. Results are expressed as percent BCC minus EV-treated cells and represent the mean ± SEM; *n* = 4; *, *P* < 0.05 compared with BCC minus EV. **F,** Flow cytometry of BCCs incubated with DiI fluorescently-labeled BCC or CSC EVs at 10^4^ EVs/cell. Results are presented as mean ± SEM; *n* = 3; *, *P* < 0.05. **G,** Representative immunofluorescence images of A549 BCCs incubated with DiI fluorescently-labeled BCC or CSC EVs.

CSCs have previously been shown to secrete EVs that affect the behavior of non-CSCs in a tumor, including tumor cells and other cells in the tumor microenvironment (37). Therefore, it is possible that the maintenance of a stem-like cell population could be driven by EV-mediated communication between CSCs and BCCs. Thus, we assessed the effects of NSCLC CSC-derived EVs on the transformed behavior of NSCLC BCCs. Single BCCs were plated in low adherence 96 wells in the presence of BCC- or CSC-derived EVs and monitored for clonal expansion. Non–EV-treated BCCs exhibited a lower cloning efficiency and produced significantly smaller spheroids when compared with non–EV-treated CSCs (Fig. 6A–C). Addition of CSC-derived EVs to BCCs resulted in a dose-dependent and significant increase in both cloning efficiency and sphere size (Fig. 6A–C). We also assessed the potential effect of BCC-derived EVs on BCC expansion. We observed a trend toward an increase in BCC cloning efficiency and expanded sphere size in low adherence cultures when BCCs were treated with higher amounts of BCC-derived EVs, but these results did not reach statistical significance (Fig. 6A–C).

CSCs may also modulate nearby BCCs into a more invasive phenotype by releasing EVs with specific cargo contents. Consistent with published reports (38), non–EV-treated control CSCs invaded 2-fold more than non–EV-treated BCCs (Fig. 6D and E). The treatment of BCCs with CSC-derived EVs increased invasion by 1.65-fold, while BCC-derived EVs had no impact on BCC invasion (Fig. 6D and E).

We next sought to determine whether these observed differences were dependent on the ability of BCCs to internalize BCC and CSC EVs. BCCs were incubated with equivalent DiI-labeled CSC- or BCC-derived EVs in cell culture medium depleted of exogenous EVs. Flow cytometry analysis revealed that A549 and H1299 BCCs internalized 2.9- and 4-fold more CSC-derived EVs compared with BCC EVs (Fig. 6F). Furthermore, BCCs incubated with DiI-labeled CSC EVs displayed a moderate but significant increase in fluorescence intensity when compared with BCCs incubated with BCC EVs (Supplementary Fig. S5), suggesting that both the percentage of BCCs that take up EVs, and the number of EVs taken up by BCCs, is greater for CSC EVs then BCC EVs. The cellular uptake of EVs by BCCs was further examined by confocal immunofluorescence microscopy, which revealed a greater number of BCCs incubated with CSC EVs displayed DiI fluorescence when compared with BCCs incubated with BCC-derived EVs. In accordance with previous reports (39), both BCC and CSC EVs were found clustered in perinuclear regions of BCCs (Fig. 6G). Together, these observations suggest that EVs released by NSCLC CSCs are more preferentially taken up by recipient BCCs when compared with BCC-derived EVs and induce a more stem-like and invasive phenotype in BCCs.

### CSCs Require RAB27B for the Transfer of EVs That Induce Stem-like Behavior in BCCs

**FIGURE 7:**
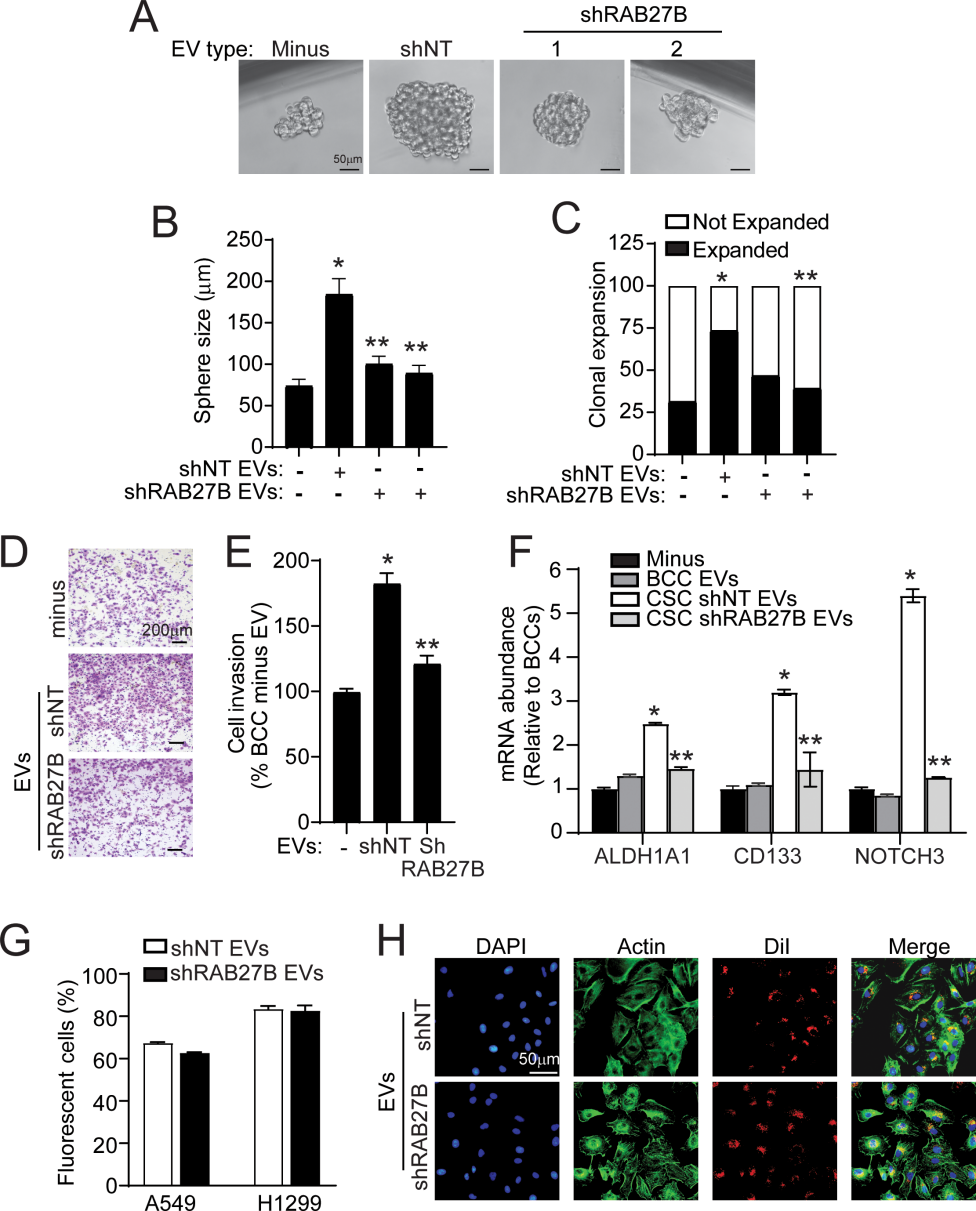
CSCs require RAB27B for the transfer of EVs that induce stem-like behavior in BCCs. **A,** Representative photomicrographs of expanded BCC spheres treated with *shNT* or *shRAB27B* CSC EVs in low-attachment culture. **B,** Quantitation of expanded sphere size (*n* = 30, *, *P* < 0.05 compared with BCC minus EV and *, *P* < 0.05 compared to *shNT* CSC-derived EV treated). **C,** Quantitation of the percent of single cell that expanded or did not expand under each condition (*n* = 30; *, *P* < 0.05 compared with BCC minus EV, **, *P* < 0.05 compared with *shNT* CSC-derived EV treated). Representative photomicrographs (**D**) and quantitation of A549 BCC invasion (**E**) through Matrigel-coated chambers with *shNT* or *shRAB27B* CSC-derived EV treatment (*, *P* < 0.05 compared with BCC minus EV, **, *P* < 0.05 compared with *shNT* CSC-derived EV treated). **F,** qPCR for ALDH1A1, CD133, and NOTCH3 mRNA abundance in A549 BCC treated with *shNT* or *shRAB27B-2* CSC-derived EVs (*n* = 3, *, *P* < 0.05 compared with BCC minus EV and **, *P* < 0.05 compared with *shNT* CSC-derived EV treated). **G,** Flow cytometry of BCCs incubated with DiI fluorescently labeled *shNT* and *ShRAB27B* CSC EVs at 10^4^ EVs/cell. **H,** Representative immunofluorescence images of A549 BCCs incubated with DiI fluorescently labeled *shNT* and *shRAB27B* CSC EVs. In B, E, F, and H, results are presented as mean ± SEM.

Next, we assessed whether RAB27B is required for the observed induction of stem-like phenotypes in BCCs treated with CSC-derived EVs. Single A549 BCCs plated in low adherence conditions in the presence of A549 *shNT* or *shRAB27B* CSC-derived EVs were monitored for clonal expansion and spheroid growth. As demonstrated in Fig. 6A–C, non–EV-treated BCCs showed lower cloning efficiency, and produced smaller spheroids than *shNT* CSC-derived EV-treated BCCs. *shRAB27B* CSC-derived EV-treated BCCs did not exhibit the increase in cloning efficiency and sphere size that was observed in *shNT* CSC-derived EV-treated cells (Fig. 7A–C). Similarly, *shNT* CSC-derived EV-treated BCCs exhibited a significant increase in cell invasion compared with untreated BCCs whereas EVs derived from *shRAB27B* CSCs failed to induce invasion in BCCs (Fig. 7D and E).

We next assessed whether the increased spheroid growth and invasion induced in BCCs by *shNT* CSC-derived EVs was accompanied by an increase in stemness-associated genes. qPCR analysis revealed that BCCs treated with BCC-derived EVs showed no significant difference in expression of stemness genes compared with non-EV treated BCCs (Fig. 7F). BCCs treated with *shNT* CSC-derived EVs expressed higher levels of ALDH1A1, CD133, and NOTCH3 when compared with BCCs treated with BCC-derived EVs. Interestingly, BCCs treated with *shRAB27B* knockdown CSC-derived EVs did not have increased expression of these genes (Fig. 7F). Similar results were observed in H1299 cells (Supplementary Fig. S6).

Given that *shRAB27B* knockdown CSC-derived EVs did not induce a stem-like phenotype in BCCs, we assessed whether this was due to differences in EV uptake. Flow cytometry analysis revealed no significant difference in A549 and H1299 BCC internalization of DiI-labeled *shNT* and *shRAB27B* CSC-derived EVs (Fig. 7G). Immunofluorescence microscopy confirmed BCC uptake of DiI-labeled *shNT* and *shRAB27B* knockdown CSC-derived EVs which exhibited similar perinuclear clustering (Fig. 7H). Taken together, our data suggest that RAB27B is required to produce CSC-derived EVs that, upon BCC internalization, can induce phenotypes associated with an aggressive stem-like phenotype, including induced expression of stemness genes, clonal expansion, spheroid growth, and invasion.

## Discussion

It is widely recognized that tumors consist of heterogeneous subpopulations of cells among which are stem-like cells (namely, CSCs) that significantly contribute to cancer initiation and recurrence, resistance to most therapies, and metastasize in advanced cancer progression. However, the mechanisms utilized by CSCs to confer these malignant phenotypes to tumors remain poorly understood. We previously conducted RNA-seq of NSCLC CSCs and BCCs and identified genes that are differentially expressed between these two cell populations (6). Intriguingly, we found that *RAB27B* was among the significantly upregulated genes in the CSC population. Here, we show that RAB27B plays a required role in the enhanced tumorigenic potential of stem-like cells isolated from NSCLC cells. Our data also provide evidence that increased RAB27B expression in CSCs is required for elevated release of EVs by CSCs with important implications in the transfer of EVs from CSCs to BCCs in NSCLC.

RAB27B protein expression has been reported to associate with progression of a variety of human cancers including ovarian, liver, colorectal, breast and the two major forms of NSCLC, LUAD, and LUSC (40–45). One study reported that RAB27B protein expression was found to be higher in NSCLC tumors with metastatic spread and higher RAB27B expression associated with poor survival rate in LUSC (42). Another study identified that RAB27B expression correlated to lymph node metastasis and poor prognosis in patients with LUAD (45). However, whether the increased expression of RAB27B in NSCLC tumors is functionally important has not been well explored. NSCLC progression has been attributed to stem-like properties of cancer cells; therefore, we sought to establish whether RAB27B plays a major role in the promotion of these properties. Our results show that RAB27B is overexpressed in CSC-enriched cultures from a panel of NSCLC cell lines and is required for enhanced transformed growth, clonal expansion, and invasion *in vitro*, and for tumor growth *in vivo*. RAB27B expression is also elevated in CSCs isolated from primary human and mouse NSCLC cells, suggesting that RAB27B may function in the maintenance of these CSCs.

RAB27B is important in secretory vesicle regulation with major functions in vesicle maturation, transport, docking, and fusion to the cell membrane. RAB27B is reported to be necessary for EV-mediated communication between cancer cells, which contributes to cancer progression and metastasis (46). Our results indicate that CSCs likely induce RAB27B to secrete elevated levels of EVs, which modulate the surrounding BCCs. It is interesting to note that A549 cells, which express RAB27B protein more abundantly than H1299 cells (Fig. 1C), also secrete more EVs than H1299 cells (Fig. 4), identifying an additional link between RAB27B abundance and the level of secreted EVs. We also observed an increase in the uptake of CSC-derived EVs by BCCs compared with BCC-derived EVs that was not dependent on RAB27B expression in the CSCs. These data suggest that the increased expression of RAB27B, while important for the elevated number of EVs secreted by CSCs, may not influence the uptake of CSC-derived EVs by BCCs.

Cancer cell–derived EVs have been shown to be potent regulators of tumor progression and resistance to therapy by transferring their cargo (proteins, lipids, mRNA, miRNA) into recipient cells, promoting signaling cascades and epigenetic changes. One study used high-resolution intravital imaging in combination with a Cre recombinase-based method to study EV exchange *in vivo* (47). The Cre-LoxP system was used to induce a color switch specifically in reporter-expressing cells that take up EVs released from cells expressing the Cre recombinase. They showed that EVs released by malignant tumor cells are taken up by less malignant tumor cells located at the secretion site and within distant tumors and that these EVs carry mRNAs involved in migration and metastasis. Other studies have also demonstrated that CSC-derived EVs could influence resident BCCs and induce a stemness phenotype that leads to tumor development and metastasis (37, 48). In line with these studies, we found that CSC-derived EVs induced expression of stemness genes, invasion, and stem-like expansion of BCCs indicating that CSC-derived EVs may carry cargo molecules which can induce molecular and phenotypic changes in non-CSCs (BCCs) that promote an aggressive stem-like phenotype. These findings further support the premise that uptake of CSC-derived EVs by BCCs increases the malignant behavior of tumors. Because it is widely accepted that the molecular composition of EVs mirrors the specialized functions of the parental cells of origin (49), future mechanistic studies are needed that focus on defining CSC-derived EV cargo and the downstream signaling that they elicit to alter the phenotype of BCCs.

Our EV uptake experiments demonstrate that BCCs preferentially internalize CSC-derived EVs compared with BCC-derived EVs. Our findings are line with a study that reported that although cancer cells can internalize EVs from nonmalignant cells, such transfer does not seem to happen frequently (47). In contrast, nonmalignant cells could readily uptake malignant cancer cell–derived EVs. Importantly, they showed that upon uptake of malignant tumor cell-derived EVs, less malignant tumor cells display enhanced migratory behavior and metastatic capacity observed in the parental line of the malignant tumor EVs. Another study reported that pancreatic ductal adenocarcinoma tumors establish an organized communication network between subpopulations of cancer cells using EVs (11). They demonstrated that communication within the network is led by CSC EVs and EVs preferentially flow from CSCs to BCCs. They found that the route of communication in the EV network is not determined by the number of secreted EVs by CSCs or BCCs, suggesting that the enhanced uptake of CSC-derived EVs by BCCs is a regulated process that could be linked to specific CSC EV cargo. We did not observe significant changes in BCC uptake of EVs derived from *shRAB27B* knockdown CSCs. However, our data show that unlike *shNT* control CSC-derived EVs, *shRAB27B* knockdown CSC-derived EVs do not increase expression of stemness markers, clonal efficiency, sphere size, and cell invasion, suggesting that the stem-like phenotypic changes in BCCs induced by CSC-derived EVs are dependent on RAB27B. Interestingly, we found that CSC-derived EVs contain more RAB27B than BCC EVs. Whether the RAB27B packaged as cargo in CSC-derived EVs directly plays a role in the ability of CSC-derived EVs to induce in a stem-like phenotype in BCCs requires further investigation. Specifically, whether the increased level of RAB27B found packaged in CSC-derived EVs (Fig. 5G) influences the loading of specific biomolecules that may contribute to the conversion of BCCs to more stem-like cancer cells warrants further study. Future lines of investigation to assess RAB27B-dependent cargo in NSCLC CSC-derived EVs may provide further mechanistic insight on the role of RAB27B in CSC biology.

CSCs have been proposed to use both cell-autonomous and non-cell autonomous mechanisms to initiate and maintain tumors. One well-reported non-cell autonomous mechanism is through CSC paracrine activities that establish communication networks between CSCs and non-CSC populations of tumors. However, the mechanisms that govern the exchange of signals between CSCs and their surrounding microenvironment need to be further explored, as these may be exploited for developing therapeutic strategies to target CSCs for cancer therapy. Our data provide evidence that the role of RAB27B in NSCLC CSCs is both cell- and non-cell autonomous. Consistent with CSCs driving tumor initiation in progression, we found that RAB27B-deficient tumors show reduced growth. Mechanistically, our results suggest that RAB27B fuels tumor cell proliferation and angiogenesis. Given that our results demonstrate that NSCLC CSCs may release EVs that promote conversion of BCCs to stem-like cells, we cannot rule out that the decreased cell invasion *in vitro*, and tumor proliferation *in vivo* exhibited by *shRAB27B* knockdown cells are not due to loss of EV-dependent communication between the CSCs and their surrounding microenvironment. In this regard, in tumor growth experiments, *shRAB27B* knockdown tumors exhibit decreased CD31 expression, a marker of angiogenesis, suggesting that RAB27B-depleted cells are deficient in providing paracrine proangiogenic signals needed to establish tumor vasculature. Our *in vitro* results suggest that the induced expression of RAB27B in CSCs may also support the autonomous growth of CSCs. This is specifically evident in our results demonstrating that knockdown of RAB27B in the CSCs reduced their expression of stemness markers and blocked their ability to clonally expand in single-cell assays *in vitro*. Additional future studies are needed to determine the molecular mechanisms that contribute to RAB27B-mediated CSC proliferation.

Overall, the key outcome of our study provides a mechanism by which CSCs produce elevated levels of EVs through upregulation of RAB27B to mediate communication between CSCs and BCCs that maintains a stem-like phenotype in NSCLC cells. One of the caveats of our study is the possibility that our data implicating RAB27B in CSC function could be influenced by the methods that we used to isolate CSCs. Nonetheless, RAB27B silencing decreased cancer stemness in each CSC function investigated in our study. Our data could be strengthened by further studies on patient samples that correlate RAB27B levels with EV secretion and CSC function. Future experiments will need to be designed to explore this avenue. Finally, EVs have been identified as a therapeutic target in cancer because their cargos have been shown to direct a variety of oncogenic functions, including cell proliferation, cell motility, metastasis, tumor angiogenesis, and chemosensitivity (37). CSCs have also emerged as an attractive therapeutic target particularly for advanced therapy-resistant cancers. Further experimental studies are required to determine whether RAB27B can be an effective target for NSCLC treatment.

## Supplementary Material

Supplementary Figure S1 Supplementary Figure S1: RAB27B expression in CSC cultures

Supplementary Figure S2 Supplementary Figure S2: RAB27B is required for the stem-like phenotype of NSCLC CSCs

Supplementary Figure S3 Supplementary Figure S3: RAB27B is required for NSCLC tumorigenicity in vivo

Supplementary Figure S4 Supplementary Figure S4: Characterization of RAB27B knockdown NSCLC CSC derived EVs

Supplementary Figure S5 Supplementary Figure S5: Cellular uptake of DiI-labeled BCC and CSC EVs

Supplementary Figure S6 Supplementary Figure S6: Effects of ShRAB27B CSC-derived EVs on the expression of stemness genes in BCCs

Supplementary Table S1 Supplementary Table S1: Primers and shRNAs used in Experimental Procedures
