# Supplementary material for: RAB27B Drives a Cancer Stem Cell Phenotype in NSCLC Cells Through Enhanced Extracellular Vesicle Secretion
Source: Cancer Res Commun. 2023 Apr 17;3(4):607–20. doi: 10.1158/2767-9764.CRC-22-0425 (PMC10109210; doi:10.1158/2767-9764.CRC-22-0425)
Supplement: Supplementary Figure S1 — RAB27B expression in CSC cultures [file crc-22-0425-s01.pdf]

Supplementary Fig. S1

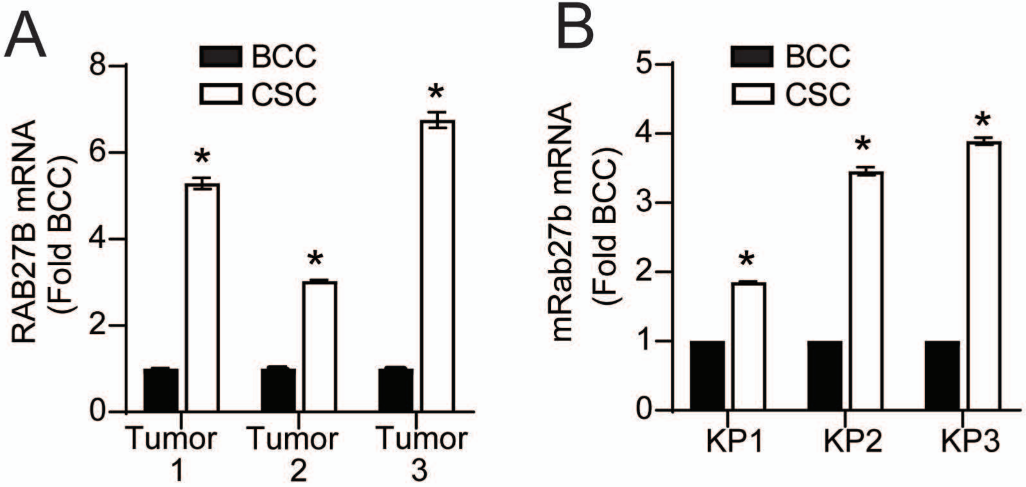

Supplementary Fig. S1. RAB27B expression is elevated in CSC cultures isolated from primary human NSCLC cases and *KrasG12D;Trp53* (KP) mouse LUADs. Quantitative real-time PCR analysis of RAB27B expression in BCCs and CSCs from **(A)** surgically resected primary human non-small cell (NLCSC) tumors and **(B)** mouse KP LUAD tumors. Results are presented as mean ± SEM, n = 3, *p < 0.05.
