# Supplementary material for: RAB27B Drives a Cancer Stem Cell Phenotype in NSCLC Cells Through Enhanced Extracellular Vesicle Secretion
Source: Cancer Res Commun. 2023 Apr 17;3(4):607–20. doi: 10.1158/2767-9764.CRC-22-0425 (PMC10109210; doi:10.1158/2767-9764.CRC-22-0425)
Supplement: Supplementary Figure S2 — RAB27B is required for the stem-like phenotype of NSCLC CSCs [file crc-22-0425-s02.pdf]

Supplementary Fig. S2

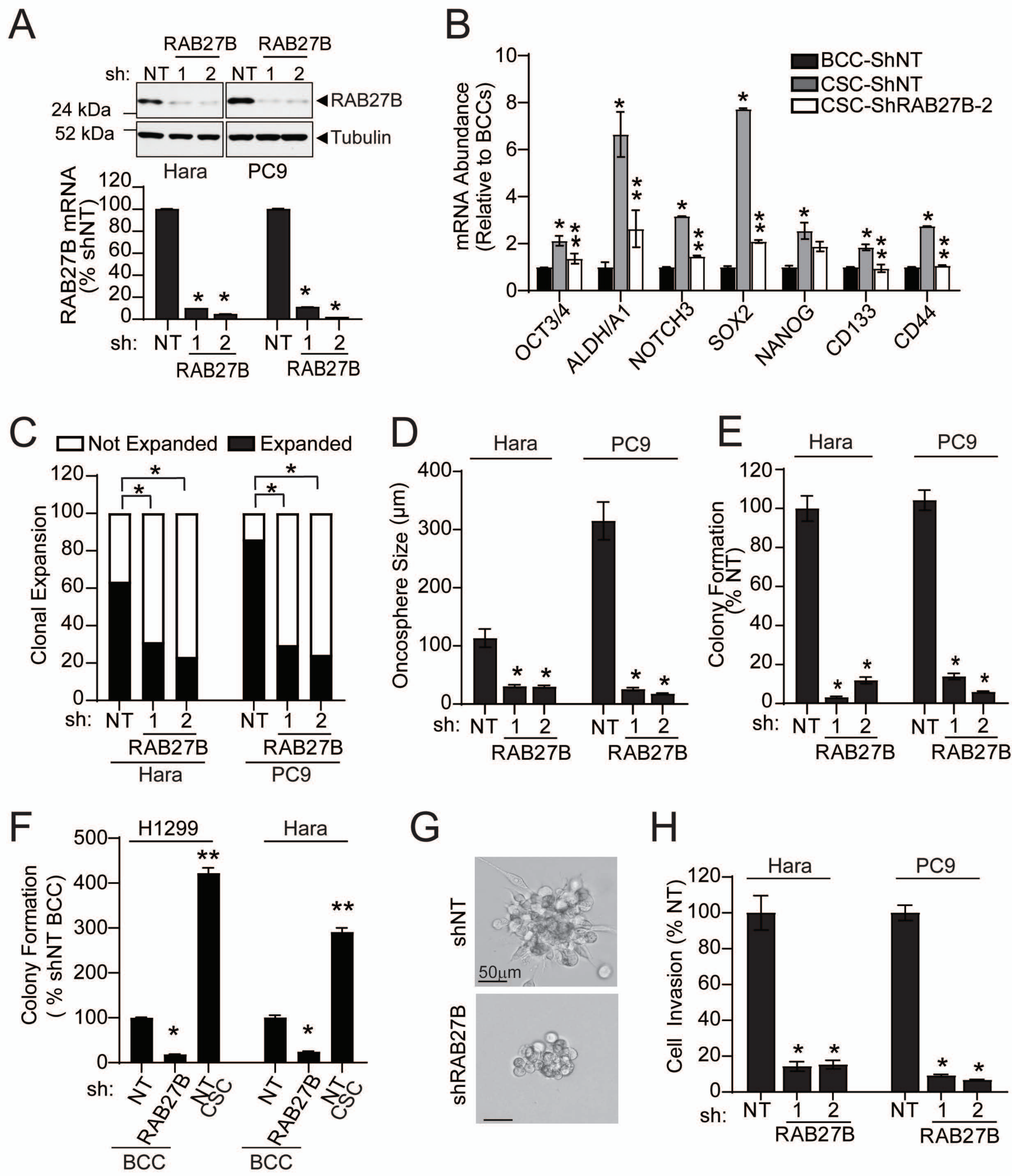

Supplementary Fig. S2. RAB27B is required for the stem-like phenotype of NSCLC CSCs. (A) QPCR and immunoblot analysis of RAB27B mRNA and protein abundance, respectively in Hara and PC9 CSCs, n = 3. QPCR analysis of RAB27B mRNA abundance in H1299 and A549 *shRAB27B* knockdown CSCs **(B)** QPCR for stem cell markers in H1299 *shNT BCC*, and *shNT* and *shRAB27-2* CSCs. Represented as mean ± SEM, n = 3, *p<0.05 *shNT* BCC vs. *shNT* CSC and **p <0.05 *shNT* CSC vs *shRAB27-2*. (**C**). Clonal expansion efficiency of Hara and PC9 *shNT* and *shRAB27B* CSCs in non-adherent cultures. Results presented as % of CSCs that expanded or did not expand, *$p < 0.05$ compared with *shNT*, n = 42 (Hara) and 37 (PC9). **(D)** Sphere size of expanded CSCs expressed as mean diameter in micrometers ± SEM, n = 42 (Hara), and 37 (PC9), *$p < 0.05$ compared to *shNT*. **(E)** Quantitation of soft agar colonies formed by Hara and PC9 *shNT* and *shRAB27B* CSCs. Results are expressed as % *shNT* control and represent the mean ± SEM, n = 5, *$p < 0.05$ compared to *shNT* control. **(F)** Soft agar colony formation in H1299 and Hara *shNT* and *shRAB27B* BCC cells. Results are expressed as % *shNT* BCC and represent the mean ± SEM, n = 5, *$p < 0.05$ compared to *shNT* BCC. **(G)** Representative photomicrographs of *shNT* and *shRAB27B* H1299 CSC spheres embedded in three-dimensional Matrigel. **(H)** Hara and PC9 *shNT* and *shRAB27B* CSC cellular invasion through Matrigel-coated chambers. Results are expressed as % NT control and represent the mean ± SEM, n = 4, *$p < 0.05$ compared to *shNT* control.
