# Supplementary material for: RAB27B Drives a Cancer Stem Cell Phenotype in NSCLC Cells Through Enhanced Extracellular Vesicle Secretion
Source: Cancer Res Commun. 2023 Apr 17;3(4):607–20. doi: 10.1158/2767-9764.CRC-22-0425 (PMC10109210; doi:10.1158/2767-9764.CRC-22-0425)
Supplement: Supplementary Figure S3 — RAB27B is required for NSCLC tumorigenicity in vivo [file crc-22-0425-s03.pdf]

Supplementary Fig. S3

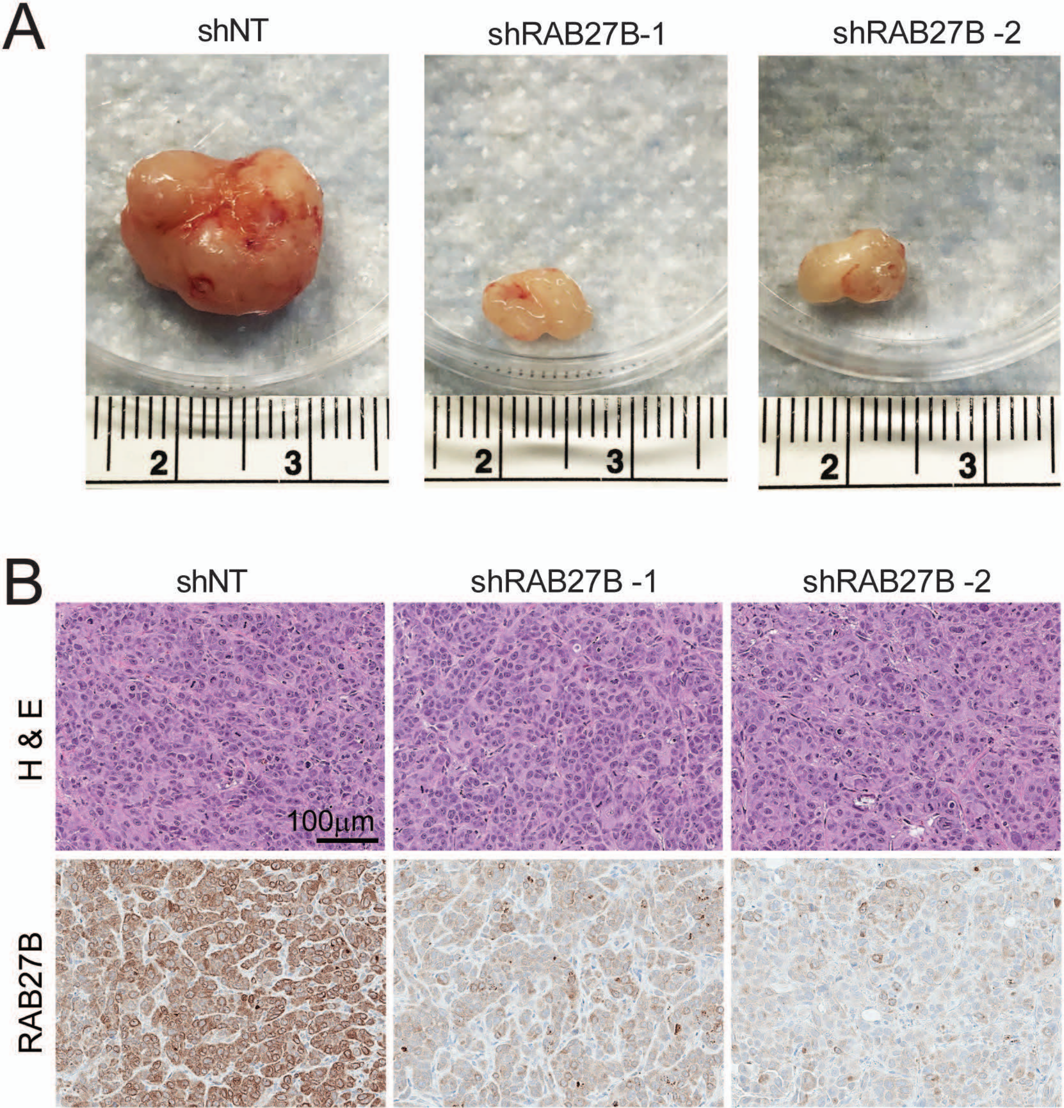

Supplementary Fig. S3. RAB27B is required for NSCLC tumorigenicity *in vivo*. (A) Photographs of representative tumors taken at time of harvest. **(B)** PC9 *shNT* and *shRAB27B* tumors stained with hematoxylin and eosin (H & E) or antibody against RAB27B by immunohistochemistry.
