# Supplementary material for: RAB27B Drives a Cancer Stem Cell Phenotype in NSCLC Cells Through Enhanced Extracellular Vesicle Secretion
Source: Cancer Res Commun. 2023 Apr 17;3(4):607–20. doi: 10.1158/2767-9764.CRC-22-0425 (PMC10109210; doi:10.1158/2767-9764.CRC-22-0425)
Supplement: Supplementary Figure S4 — Characterization of RAB27B knockdown NSCLC CSC derived EVs [file crc-22-0425-s04.pdf]

Supplementary Fig. S4

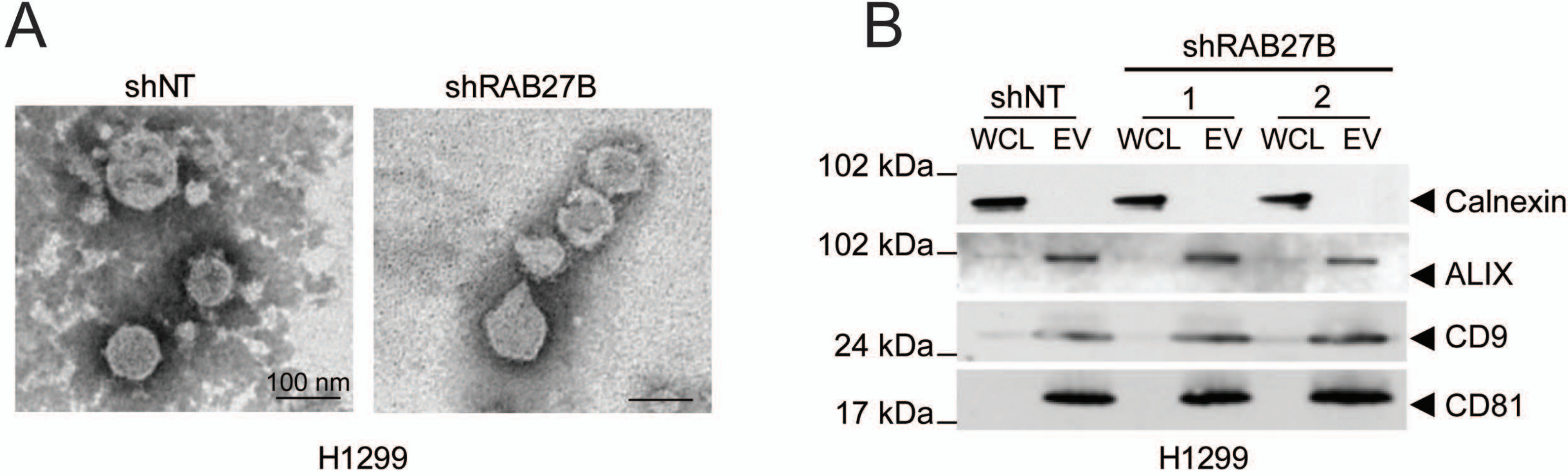

Supplementary Fig. S4. RAB27B knockdown characterization of NSCLC CSC derived EVs. **(A)** Transmission electron microscopy images showing the particle size and spherical vesicle morphology of H1299 *shNT* and *shRAB27B-2* CSC-derived EVs (100 nm). **(B)** Immunoblot analysis of calnexin, ALIX, CD9, and CD81 in the whole cell lysate (WCL) and EVs of H1299 *shNT* and *shRAB27B* CSC.
