# Supplementary material for: RAB27B Drives a Cancer Stem Cell Phenotype in NSCLC Cells Through Enhanced Extracellular Vesicle Secretion
Source: Cancer Res Commun. 2023 Apr 17;3(4):607–20. doi: 10.1158/2767-9764.CRC-22-0425 (PMC10109210; doi:10.1158/2767-9764.CRC-22-0425)
Supplement: Supplementary Figure S5 — Cellular uptake of DiI-labeled BCC and CSC EVs [file crc-22-0425-s05.pdf]

Supplementary Fig. S5

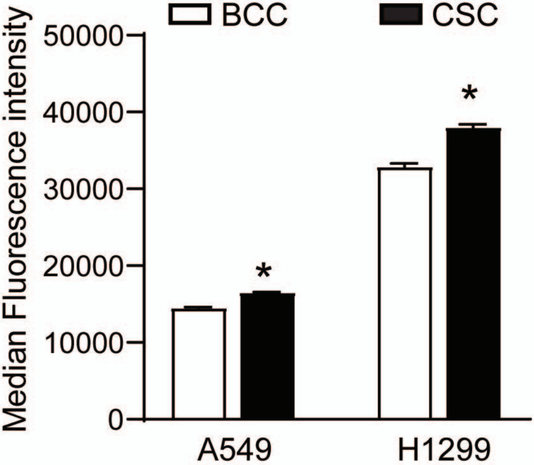

Supplementary Fig. S5. Cellular uptake of DiI-labeled BCC and CSC EVs. Median fluorescence intensity of DiI in A549 and H1299 BCCs measured by flow cytometry. Results are presented as mean ± SEM. N=3, *$p < 0.05$.
