# Supplementary material for: RAB27B Drives a Cancer Stem Cell Phenotype in NSCLC Cells Through Enhanced Extracellular Vesicle Secretion
Source: Cancer Res Commun. 2023 Apr 17;3(4):607–20. doi: 10.1158/2767-9764.CRC-22-0425 (PMC10109210; doi:10.1158/2767-9764.CRC-22-0425)
Supplement: Supplementary Figure S6 — Effects of ShRAB27B CSC-derived EVs on the expression of stemness genes in BCCs [file crc-22-0425-s06.pdf]

Supplementary Fig. S6

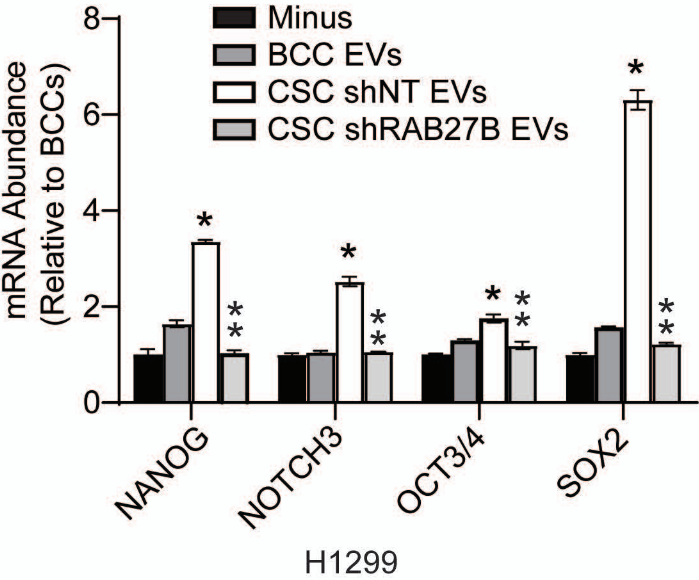

Supplementary Fig. S6. Effects of *ShRAB27B* CSC-derived EVs on the expression of stemness genes in BCCs. QPCR for NANOG, NOTCH3, OCT3/4 and SOX2 mRNA abundance in H1299 BCC treated with *shNT* or *shRab27B-2* CSC-derived EVs. n = 3, *p<0.05 vs. BCC minus EV and **p <0.05 vs. *shNT* CSC-derived EV treated. Results are presented as mean ± SEM.
