# Supplementary material for: RAB27B Drives a Cancer Stem Cell Phenotype in NSCLC Cells Through Enhanced Extracellular Vesicle Secretion
Source: Cancer Res Commun. 2023 Apr 17;3(4):607–20. doi: 10.1158/2767-9764.CRC-22-0425 (PMC10109210; doi:10.1158/2767-9764.CRC-22-0425)
Supplement: Supplementary Table S1 — Primers and shRNAs used in Experimental Procedures [file crc-22-0425-s07.pdf]

Supplementary Table S1

A. Real-time qPCR Probes

Target	ABI Assay ID
human RAB27B	Hs00188156
mouse RAB27B	Mm0122252
RAB27A	Hs00608302
human UBC	Hs00824723
mouse UBC	Mm02525934
human OCT3/4	Hs04260367
human ALDH1A	Hs009746916
human NOTCH3	Hs01128541
human SOX2	Hs04234836
human NANOG	Hs02387400
human CD133	Hs 01009259
human CD44	Hs01075864

B. shRNA Targeting Sequence

RAB27B	shRNA 1: CCAGTCAACAGAGCTTCTTAA<br>shRNA 2: GATACTGTCAATGGTGGAAAT
Non-target	Sigma Aldrich-SHC016

Supplementary Table S1: Primers and shRNAs used in Experimental Procedures. A) Primer probe sets for real time qPCR. Designated ABI Assay IDs are listed. **B)** shRNA target sequences
